# Postpartum depression in mothers and fathers: a structural equation model

**DOI:** 10.1186/s12884-020-03228-9

**Published:** 2020-09-15

**Authors:** Zhizhou Duan, Yuanyuan Wang, Ping Jiang, Amanda Wilson, Yan Guo, Yongliang Lv, Xiaonan Yang, Renjie Yu, Shuilan Wang, Zhengyan Wu, Mengqing Xia, Guosheng Wang, Ye Tao, L Xiaohong, Ling Ma, Hong Shen, Jue Sun, Wei Deng, Yong Yang, Runsen Chen

**Affiliations:** 1 Institute of Mental Health, Suzhou Guangji Hospital, Affiliated Guangji Hospital of Soochow University, Soochow University, No. 11 Guangqian Road, Jiangsu Province 215137 Suzhou, PR China; 2 School of Health Sciences, Wuhan university, 430072 Wuhan, Hubei Province China; 3 National Clinical Research Center for Mental Disorders, Department of Psychiatry, and China National Technology Institute on Mental Disorders, The Second Xiangya Hospital of Central South University, 410011 Changsha, Hunan China; 4 Division of Psychology, Faculty of Health and Life Sciences, De Montfort University, Leicester, UK; 5 Suzhou science & technology town hospital, Suzhou, China; 6 Gusu District Wumenqiao Street Canglang Xincheng Community Health Service Center, Suzhou, China; 7 Mental Health Center, West China Hospital of Sichuan University, Chengdu, PR China

**Keywords:** paternal post-partum depression, maternal post-partum depression, mother-in-law relationship, marital satisfaction, Sturctural Equation Modelling (SEM)

## Abstract

**Background:**

Post-partum depression (PPD) is a growing mental health concern worldwide. There is little evidence in the Chinese context of the relationship between paternal PPD and maternal PPD. Given the growing global concerns this relationship requires further exploration.

**Methods:**

A survey was conducted with 950 total couples from March 2017 to December 2018. The study was conducted using a standardized questionnaire that included basic demographic information, information on the relationship between the mother-in-law and daughter-in-law, marital satisfaction (both maternal and paternal), and PPD symptoms. Structural Equation Modelling (SEM) analysis was used to explore the underlying mechanism for PPD symptoms in mothers and fathers.

**Results:**

In 4.4% of the couples both the wife and the husband showed depressive symptoms. Maternal marital satisfaction showed a significant mediating effect on paternal PPD (B = -0.114, *p* < 0.01), and there was a direct effect of maternal PPD on paternal PPD (B = 0.31, *p* < 0.001).

**Conclusions:**

This is the first study to investigate the possible correlation between maternal PPD, mother-in-law and daughter-in-law relationship satisfaction, maternal marital satisfaction, paternal marital satisfaction, and paternal PPD. It is important for future PPD interventions to target both maternal and paternal mental health, as well as the mechanisms identified that can lead to PPD.

## Background

Post-partum depression (PPD) is a growing mental health concern and one of the leading causes of poor familial and infant health outcomes [1–3]. Similar to maternal PPD, PPD can also effect the mental health of fathers [4]. Paternal PPD refers to a new father who suffers from depressive symptoms within a 12 month perinatal period after the birth of an infant [5, 6]. Paternal PPD has a negative impact on family members, the impact can reduce early parent-infant interactions resulting in poor communication and stimulation [7].It can also have further long-term adverse impacts on an infant’s physical growth and their cognitive, behavioral, and social development [8, 9]. Previous research has shown the rate of paternal PPD ranges from 5.4–13.6%, depending on sample size, measurement scales, and different population characteristics [4, 10–12]. The high rate of paternal PPD has received worldwide concern [4], and research should be conducted to provide an evidence base to this timely subject.

To date, several risk factors of paternal PPD have been identified including maternal PPD, marital satisfaction, and social support [11, 13–15]. The state of a new mother’s perinatal mental health has been found to influence paternal PPD [14, 16]. The rate of paternal PPD is also significantly associated with rates of maternal PPD [4, 17].If there is maternal PPD this has been found to be the strongest direct predictor of paternal PPD, which may be explained by the couple’s adjustment within a new stage of parenthood where changes in factors of partner support and relationship satisfaction can impact on the mood of both new mothers and fathers, increasing their risk for depression [16].

Paternal PPD has also been strongly associated with paternal marital satisfaction. A previous study has indicated that marital satisfaction was one of the most important predictors for paternal PPD [4]. According to a longitudinal study surveyed in Hong Kong, an inharmonious marriage can increase the risks of paternal PPD [18]. In addition, previous research has found that the mother-in-law relationship can largely decrease the level of paternal marital satisfaction [19]. According to a study in China, maternal marital satisfaction also has a large impact on paternal marital satisfaction, meaning those with higher maternal marital satisfaction are more likely to have greater levels of paternal marital satisfaction [20].

In addition, a good relationship between the mother-in-law and daughter-in-law is positively correlated with a higher degree of maternal marital satisfaction [21]. And in turn, the women experiencing postpartum depression can aggravate the relationship with the mother-in-law, negatively impacting marital satisfaction further [18]. Within Chinese culture, the relationship with the mother-in-law and maternal marital satisfaction are closely linked to maternal PPD[2, 18, 22]. A poor relationship with the mother-in-law and maternal marital satisfaction can therefore significantly increase the risk of maternal PPD [2].

Unlike maternal PPD, there are insufficient studies focused on PPD among new fathers to further understand the factors and associations of paternal PPD. Therefore, we aimed to use structural equation modeling (SEM) to explore the relationship among maternal PPD, mother-in-law relationship satisfaction, maternal marital satisfaction, paternal marital satisfaction, and paternal PPD. We hypothesized that (1) maternal marital satisfaction and mother-in-law relationship satisfaction could indirectly impact on paternal PPD via paternal marital satisfaction; (2) maternal PPD, mother-in-law relationship satisfaction, and maternal marital satisfaction would be associated.

## Methods

### Participants

This cross-sectional study was conducted in 3 local community health service centers and obstetric wards in Suzhou city, China between March 2017 and December 2018 [23–25]. Participants who met the following inclusion criteria were included in the study: (1) the participants were married; (2) participants were equal or above the legal age of marriage (20 years old for women, and 22 years old for men) [26]; (3) women who have given birth in the last year; (4) both partners in the couples agreed to participate and provided informed consent. A total of 1,034 couples were recruited and 950 couples met the inclusion criteria. All data was collected by trained researchers. Following the Declaration of Helsinki, the researchers fully illustrated the purpose of the study in the information sheet, considerations were made to protect the privacy of participants through assigning them a participant number, and the information sheet informed participants of their right to withdraw at any point, including how to do so [27]. The Ethics Committee of the Suzhou Guangji Hospital in China (reference number: 2016-010) approved this study.

### Measures

#### Basic Socio-demographic characteristics

Basic Socio-demographic characteristics were collected including age, residence, whether the new father was the only child, yearly family income, educational level, and if there was any family history of psychological disorders.

#### Mother-in-law relationship

The relationship between mother-in-law and daughter-in-law was assessed using the question “Do you currently feel satisfied with the relationship with your mother-in-law?” (answered by the wife). The options on the likert scale ranged from 1 (extremely unsatisfactory) to 7 (extremely satisfactory). This question has been used in previous research to measure satisfaction of the wife within the mother-in-law relationship [2].

#### Marital Satisfaction (wife/husband)

Marital satisfaction (wife/husband) was measured by the question “Do you currently feel satisfied in your marital relationship?” (answered by both the wife and husband). The participants could respond on a scale of extremely unsatisfactory (scored 1) to extremely satisfactory (scored 7). This question has been used for marital satisfaction assessment in previous research [28].

#### Postpartum depression (wife/husband)

For both wives and husbands, PPD was measured by the Edinburgh Postnatal Depression Scale (EPDS). The EPDS is a 10-item 4-point Likert scale, with a higher total score indicating a higher risk of PPD. The cut-off score of the EPDS scale was set to 10 to indicate depressive signs [29]. A Chinese version of the EPDS was used (the Cronbach alpha ranged from 0.79 to 0.87) [30, 31]. In this study, the Cronbach alpha was 0.85 (wife) and 0.83 (husband) respectively.

### Statistical analysis

For the basic socio-demographic characteristics, we used frequencies and percentages to summarize related characteristics. A Spearman’s Correlation analysis was calculated to explore the relationship between the variables. The above analysis used SPSS (version 21.0) and the significance level was considered at *p* < 0.05(two-tailed). For the SEM analysis, we used SPSS AMOS module 20.0 to test our hypothesized model. The SEM model was applied to understand how several paths when combined could predict how maternal marital satisfaction and mother-in-law relationship satisfaction indirectly impact on paternal PPD via paternal marital satisfaction. It also looked at the association between maternal PPD, mother-in-law relationship satisfaction, and maternal marital satisfaction. The SEM model was a good fit, with indexes as follows: Comparative fit index, CFI > 0.90; Tucker Lewis index, TLI > 0.90, Goodness of fit index, GFI > 0.90; Normed fit index, NFI > 0.90; and had a root mean square error of approximation, RMSEA < 0.05 and χ2/df < 3(*P* > 0.05) [32, 33]. In the above indicies CFI, χ2/df, and absolute fit directly assess how well a model fits the observed data; RMSEA, corrects for a model’s complexity and other indices compensate for the effect of model complexity [33].

## Results

As shown in Table 1, among the total 950 couples eligible to participate, the mean age of a new father was 31.7 years old (SD = 3.93) and 52.9% of new fathers were between 31 and 35 years old. Among all new father participants, 55.3% were urban residence and 45.4% were the only child. More than half of the participants (64.7%) reported a yearly family income of more than 15 (unit in ten thousand yuan) and most of the individuals (81.2%) had a bachelor’s degree or higher. Also, 4.3% of individuals had a family history of psychological disorders, 10.2% showed depressive symptoms, and in 4.4% of the couples both the wife and husband showed depressive symptoms.

**Table 1:** Socio-demographic characteristics of new fathers

Characteristic	Number	Percent(%)
**Age**		
22–25	33	3.6
26–30	251	27.6
31–35	481	52.9
≥ 36	145	15.9
Mean, SD	31.67	3.93
**Residence**		
Rural	425	44.7
Urban	525	55.3
**Being the only child**		
Yes	431	45.4
No	519	54.6
**Yearly family income (ten thousand yuan)**		
< 15	335	35.3
15–30	483	50.9
> 30	131	13.8
**Educational level**		
High school or lower	179	18.8
Bachelor degree or higher	771	81.2
**Marital status**		
First marriage	946	99.6
Remarriage	4	0.4
**Family mental health history**		
Yes	41	4.3
No	909	95.7
**Depression symptom (new father)**		
≥ 10	97	10.2
< 10	853	89.8
**Depression symptom (couples)**		
≥ 10	42	4.4
< 10	908	95.6

In Table 2, the correlation analysis shows that paternal PPD was positively correlated with maternal PPD (*r* = 0.423, *p* < 0.01) and negatively correlated with mother-in-law relationship satisfaction (*r*=-0.141, *p* < 0.01), maternal marital satisfaction (*r*=-0.193, *p* < 0.01), and paternal marital satisfaction (*r*=-0.157, *p* < 0.01). Suggesting those with higher levels of maternal PPD, lower levels of mother-in-law relationship satisfaction, and lower levels of marital satisfaction are more likely to be correlated with having higher levels of paternal PPD. Furthermore, the five key variables were significantly correlated with each other (*p* < 0.01).

**Table 2:** Key variables and Spearman’s Correlation coefficients

	Mean	SD	1	2	3	4	5
1. Mother-in-law relationship	5.40	1.30	1				
2. Maternal marital satisfaction	5.67	1.19	0.728**	1			
3. Maternal postpartum depression	5.83	4.80	-0.168**	-0.188**	1		
4. Paternal marital satisfaction	6.14	0.95	0.307**	0.423**	-0.160**	1	
5. Paternal postpartum depression	4.07	3.99	-0.141**	-0.193**	0.423**	-0.157**	1

**p < 0.01

**Fig. 1:**
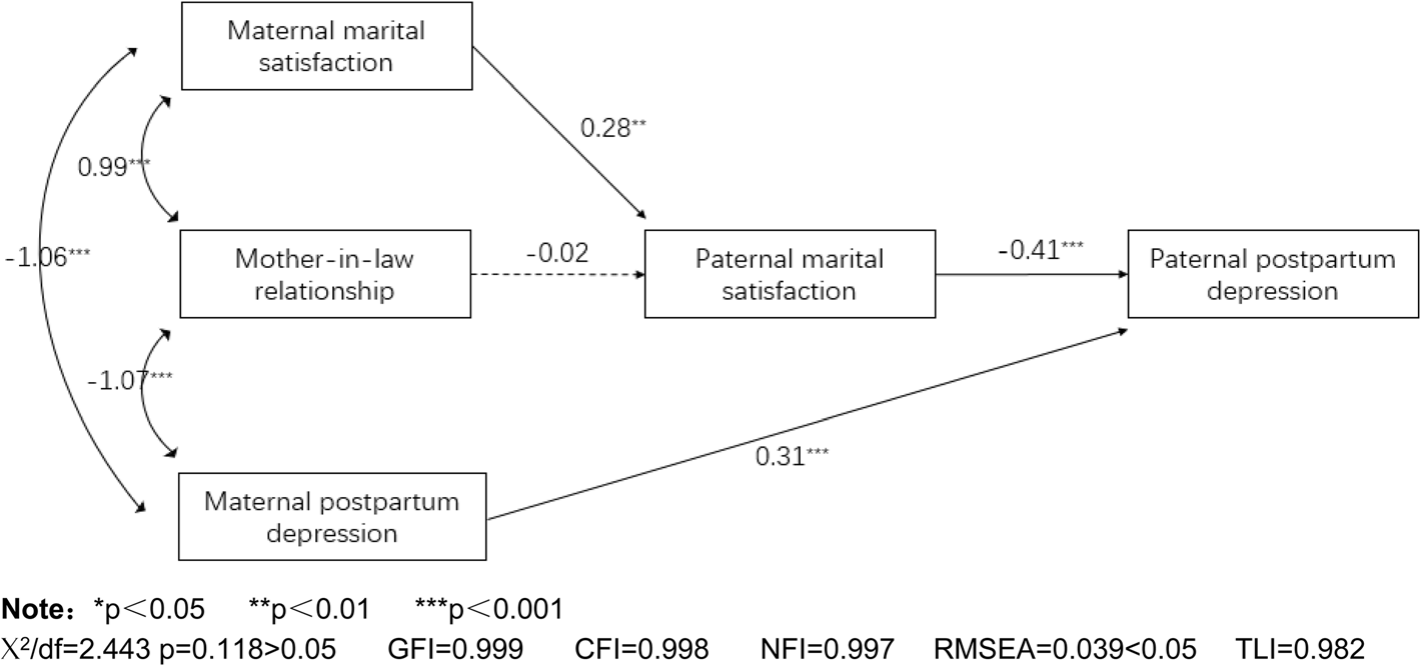
The final mediation model. Note: *p < 0.05 **p < 0.01 ***p < 0.001. X2/df=2.443 p=0.118>0.05 GFI=0.999 CFI=0.998 NFI=0.997 RMSEA=0.039<0.05 TLI=0.982.

In Fig. 1, the final SEM model shows the mechanism of maternal PPD, mother-in-law relationship satisfaction, maternal marital satisfaction, paternal marital satisfaction, and paternal PPD. The final SEM model indicated a good fit (χ^2^/df = 2.443, p = 0.118, RMSEA = 0.039, GFI = 0.999, CFI = 0.998, NFI = 0.997, TLI = 0.982). Furthermore, the final model revealed that the specific standardized effect of maternal PPD on paternal PPD was 0.31(B = 0.31, *p* < 0.001) showing that paternal marital satisfaction predicted paternal PPD, B=-0.41, *p* < 0.001, and maternal marital satisfaction predicted paternal marital satisfaction, B=-0.41, *p* < 0.001. In addition, maternal marital satisfaction, mother-in-law relationship, and maternal PPD were significantly correlated with each other in the model (maternal marital satisfaction and mother-in-law relationship, B = 0.99, *p* < 0.001; mother-in-law relationship and maternal PPD, B=-1.07, *p* < 0.001; maternal marital satisfaction and maternal PPD, B=-1.06, *p* < 0.001). All in all, the total effect of maternal marital satisfaction on paternal PPD was B= -0.11(*p* < 0.01), and maternal marital satisfaction had an indirect effect on paternal PPD by paternal marital satisfaction. The effect of mother-in-law relationship satisfaction on paternal marital satisfaction was not significant.

## Discussion

To our knowledge, this is the first study to explore the possible correlations between maternal PPD, mother-in-law relationship satisfaction, maternal marital satisfaction, paternal marital satisfaction, and paternal PPD using SEM analysis. Our research confirms previous findings in which maternal PPD plays an important role in predicting paternal PPD. In our study, our outcomes showed the prevalence of paternal PPD in the couples sampled. This rate is consistent with previous research, with a recent meta-analysis reporting similar overall rates of PPD in new fathers during the initial perinatal period [4]. Targeted towards new fathers in China, another recent meta-analysis also showed a prevalence of paternal PPD that was consistent with our study [34].

The model further showed that maternal marital satisfaction had an indirect effect on paternal PPD by paternal marital satisfaction. Therefore, our study suggests that due attention should been given to a new mothers’ condition and that signs of marital dissatisfaction should also be considered as a risk factor for paternal PPD. Screening new mothers for PPD and martial satisfaction would then create more effective interventions to identify if there is a need to screen new fathers for PPD, providing a window for early intervention for paternal PPD and familial and infant health [35]. Paternal marital satisfaction was a full mediating factor in the relationship between maternal marital satisfaction and paternal PPD. Although there is a lack of research that reports how paternal marital satisfaction can predict the development of paternal PPD, it has been supported in the previous literature, indirectly indicating that, a disharmonious marriage can further increase the risk of paternal PPD, and that furthermore maternal marital satisfaction can exert considerable influence on paternal marital satisfaction [20, 36]. As a result, our study suggests that both maternal and paternal marital satisfaction be taken into consideration as another risk factor to consider when aiming to reduce the rates of paternal PPD. If the new mother shows both signs of PPD and martial dissatisfaction, this study recommends screening of fathers not only for PPD, but also for marital dissatisfaction as well. The ability to identify paternal PPD and marital dissatisfaction earlier would have a positive impact on the child’s lifespan development, amongst other key cognitive, behavioral, and social aspects, as suggested in the literature reviewed.

In this study the mother-in-law relationship was not associated with paternal marital satisfaction, which is in contrast to other research [19]. However, the previous studies do not consider the effects of maternal marital satisfaction in conjunction with paternal marital satisfaction. Therefore, there should be caution around the assumption that a poor relationship with the mother-in-law can predicted the level of paternal marital satisfaction directly. However, further research is needed that looks at the maternal and paternal mother-in-law relationships to provide a more robust base of evidence. In our current study, mother-in-law relationship satisfaction should still receive due attention because of the potentially effect on maternal marital satisfaction and maternal postpartum depression, even though it indirectly predicted the level of paternal marital satisfaction. If a new mother is dissatisfied with the mother-in-law relationship, her dissatisfaction likely could lead to marital dissatisfaction, which this study further shows predicts paternal PPD and marital dissatisfaction.

In summary, our study indicated that the three key factors related to the wife (maternal PPD, mother-in-law relationship satisfaction, and maternal marital satisfaction) were significantly associated. These three factors can act on paternal PPD by the pathway of paternal marital satisfaction. One of the most common familial conflicts that occurs is within the relationship between the wife and mother-in-law in China, which is a key aspect of Chinese culture [2]. It can increase the family disharmony and have an important impact on marital satisfaction between a wife and husband [22], increasing the risk of paternal PPD. Therefore, more attention should focus on the family network and the relationship among family members, with the goal of forming a harmonious atmosphere within the family. Interventions should then target the whole family if there is maternal dissatisfaction with the mother-in-law relationship. This disharmony should be identified prior or during pregnancy to buffer against maternal and paternal PPD. This could be achieved by using the scales mentioned in this study during prenatal appointments.

There are some limitations to the present study. First, this study was a cross-sectional survey in one city in China, which deceases the levels of representativeness of the sample and a causal relationship cannot be fully established. Second, some of the factors influencing how women can increase the risk of paternal PPD were not surveyed due to the accessibility of couples. Third, the measure of the history of family psychological disorders and marital satisfaction were self-reported, which may cause some bias in this study. While we have tried to demonstrate the relationship between maternal PPD and paternal PPD on marital satisfaction, future studies should explore paternal PPD and other related risk factors to create more effective intervention measures.

## Conclusions

In conclusion, our study provided that the mechanism of paternal PPD that best predicted a new mother’s marital satisfaction and risk of PPD accordingly. Maternal PPD and maternal marital satisfaction were found to be central factors when reducing the prevalence of paternal PPD. Therefore, there should be considerable importance placed on seeking to improve marital satisfaction and the mother-in-law relationship satisfaction amongst the mother-in-law and the wife.

## Data Availability

The datasets used and/or analysed during the current study are available from the corresponding author on reasonable request.

## Abbreviations

PPD: Postpartum depression
SEM: Structural equation model (SEM)
EPDS: Edinburgh Postnatal Depression Scale
